# Willingness to pay for flexible working conditions of people with type 2 diabetes: discrete choice experiments

**DOI:** 10.1186/s12889-017-4903-6

**Published:** 2017-12-15

**Authors:** M. A. Nexo, B. Cleal, Lise Hagelund, I. Willaing, K. Olesen

**Affiliations:** 1 Diabetes Management Research, Steno Diabetes Center, Niels Steensens Vej 6, 2820 Gentofte, Denmark; 2 Incentive, 2840 Holte, Denmark

**Keywords:** Diabetes, Occupational health, Discrete choice experiments, Willingness to pay

## Abstract

**Background:**

The increasing number of people with chronic diseases challenges workforce capacity. Type 2 diabetes (T2D) can have work-related consequences, such as early retirement. Laws of most high-income countries require workplaces to provide accommodations to enable people with chronic disabilities to manage their condition at work. A barrier to successful implementation of such accommodations can be lack of co-workers’ willingness to support people with T2D. This study aimed to examine the willingness to pay (WTP) of people with and without T2D for five workplace initiatives that help individuals with type 2 diabetes manage their diabetes at work.

**Methods:**

Three samples with employed Danish participants were drawn from existing online panels: a general population sample (*n* = 600), a T2D sample (*n* = 693), and a matched sample of people without diabetes (*n* = 539). Participants completed discrete choice experiments eliciting their WTP (reduction in monthly salary, €/month) for five hypothetical workplace initiatives: part-time job, customized work, extra breaks with pay, and time off for medical consultations with and without pay. WTP was estimated by conditional logits models. Bootstrapping was used to estimate confidence intervals for WTP.

**Results:**

There was an overall WTP for all initiatives. Average WTP for all attributes was 34 €/month (95% confidence interval [CI]: 27–43] in the general population sample, 32 €/month (95% CI: 26–38) in the T2D sample, and 55 €/month (95% CI: 43–71) in the matched sample. WTP for additional breaks with pay was considerably lower than for the other initiatives in all samples. People with T2D had significantly lower WTP than people without diabetes for part-time work, customized work, and time off without pay, but not for extra breaks or time off with pay.

**Conclusions:**

For people with and without T2D, WTP was present for initiatives that could improve management of diabetes at the workplace. WTP was lowest among people with T2D. Implementation of these initiatives seems feasible and may help unnecessary exclusion of people with T2D from work.

## Background

The workforce of many high income countries is aging [1]. The prevalence of type 2 diabetes (T2D) is higher in older populations, and the future workforce will comprise proportionately more individuals with T2D. Moreover, the age of onset of T2D is decreasing, [2] further increasing the number of individuals with T2D who are of working age. T2D has been associated with work-related consequences such as decreased productivity [3–5] and early retirement [6, 7]. However, the mechanisms responsible for the exclusion of people with T2D from the labour market remain unexplored.

In accordance with the World Health Organisation’s model of health and disability, the interplay between biological, psychosocial, individual, and contextual factors can help explain how diabetes impacts the ability to carry out activities at work and the ability to participate in societal roles, including work [8, 9]. Low socioeconomic status has been associated with increased risk of early retirement among people with T2D [10]; job type and education level are likely individual factors that influence options for managing health-related work disability. Results from a few qualitative studies suggest that contextual factors such as support from colleagues and managers demonstrated by, for example, the possibility of frequent breaks [11–14] can make it easier to monitor blood glucose and manage dietary and medical needs at work. In contrast, stigma has been identified as a barrier to successful management of diabetes in daily life [15, 16].

In most high income countries, including Denmark, employers are legally required to undertake initiatives that enable people with chronic disabilities to manage their conditions at work; an example is the Employment Equality EU directive [17]. Initiatives entail any reasonable work accommodations, which can be any adjustments to a job, the work environment, or the organisation of work to help individuals with disabilities perform job functions, or secure equal access to benefits in the workplace [18]. Although these regulations help ensure basic rights for people with T2D, successful implementation of work accommodations are likely to be influenced by workers’ willingness to provide support for people with T2D at work. More knowledge about workers’ preferences for work initiatives can help policymakers and healthcare providers identify effective strategies to prevent future exclusion of people with T2D from the labour market.

Discrete choice methods are increasingly used to determine preferences of people with diabetes [19–21]. In a typical discrete choice experiment, participants choose between two hypothetical scenarios with different statements that often include their willingness to pay (WTP) for specific treatments. Choice probabilities analyses provide an overview of participants’ preferred choices in relation to attributes of alternatives. This study examined the WTP of individuals with and without T2D with respect to flexible working conditions for people with T2D.

## Methods

An online survey was designed and developed in the spring of 2015 in Denmark.

### Population

Individuals from web-based panels participated in the survey. The research database (http://www.userneeds.dk) includes around 150,000 individuals, who have been selected and invited to register for online surveys in return for small rewards (e.g. discounts or coupons). Individuals were included if they consented to participate, were between 25 and 67 years old, employed (full or part time) at a workplace with at least one other employee, and residing in Denmark. Three samples were formed by applying quotas based on gender, age groups and geography (Eastern and Western Denmark) and based on whether participants had identified themselves as having T2D (Self-reported information: ‘Has a physician or other healthcare professional ever told you that you had diabetes?’ Response options included yes, type 1 diabetes; yes, type 2 diabetes; no):

- participants representative of the Danish general population in terms of age, gender, and geography: Eastern and Western region (*n* = 624)
- Individuals who had identified themselves as having T2D (*n* = 720)
- Individuals who had identified themselves as not having type 1 or type 2 diabetes, and who were matched to the T2D sample by age, gender, and region (*n* = 560)

**Fig. 1:**
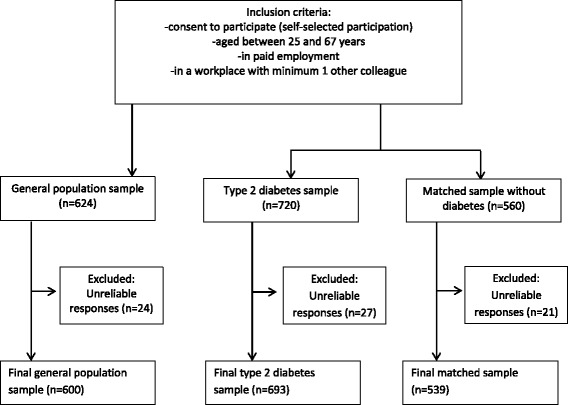
Flow diagram of the study populations

Only participants who had identified themselves as willing to participate and fulfilled the inclusion criteria were directed to the online questionnaire and completed the survey. A total of 72 respondents indicated that they had not answered the survey reliably (they reported that they ‘did not understand the questionnaire’, ‘were bored’, or ‘wanted to be done with the questionnaire’) and were excluded from the samples. The final samples therefore amounted to: General population sample = 600, T2D sample = 693, and matched sample = 539 individuals (flow diagram Fig. 1). The data collection took place from March 24 to June 10 2015.

### Survey instrument

The questionnaire included items about patient demographics, health status (e.g., use of diabetes medications, diabetes specific complications, self-rated health) and working conditions, as well as the six discrete choice experiments. The experiments were developed based on a literature review, experts in diabetes, occupational health and health economics. Four individuals with T2D recruited from a diabetes clinic in Copenhagen area evaluated the questionnaire in individual interviews. The questionnaire was pilot tested with 92 individuals (of which 33 had T2D) from web-based panels. The pilot test mainly led to modifications in pay reductions of the WTP scenarios as these were initially set to low.

The experiments were developed according to good research practices identified by Johnson et al. [22]. Each hypothetical option could be combined according to the six different workplace initiatives and attribute levels. To reduce all possible questions to a manageable number, a standardized process in the software Ngene was applied. A fractional design that was both balanced and orthogonal resulted. A balanced design ensured that the levels occurred within each attribute with equal frequency, yielding equally robust results for all levels. An orthogonal design resulted in uncorrelated parameter estimates and ensured that parameter estimates were determined independently of other attributes in the discrete choice games.

Each respondent was presented with six scenarios reflecting workplace flexibility.

In the discrete choice experiments, participants were asked to choose between two hypothetical scenarios consisting of different combinations of four workplace initiatives, each of which had an identified cost (examples shown in Table 1). The scenarios addressed the attributes of whether people with diabetes should be given the opportunity to: 1) work part-time (with pay reduction); 2) customize work by retraining for a new job or receiving a new job function; 3) take extra breaks with pay; and 4) receive time off for medical appointments or patient education during working hours with and without pay. The first three scenarios had two levels (yes/no) and the ‘receive time off’ had three (yes with pay, yes without pay; no). The attribute regarding reduction in participants’ monthly pay had four levels: 7€, 13€, 27€, and 66€ (Table 1).

**Table 1:** Overview of the six discrete choice experiments presented to the three samples

	Discrete Choice Experiment 1.		Discrete Choice Experiment 2.	
Attributes *(keywords)*	Possibility A	Possibility B	Possibility A	Possibility B
*Part-time work*	No	Yes	Yes	No
*Customized work*	No	Yes	Yes	No
*Extra breaks with pay*	Yes	No	Yes	No
*Medical visits work hours*	Yes, without pay	No	No	Yes, with pay
*Monthly pay reduction*	27 €	13 €	7 €	66 €
*Preferred option (A or B)?*	о	о	о	о
	Discrete Choice Experiment 3.		Discrete Choice Experiment 4.	
Attributes *(keywords)*	Possibility A	Possibility B	Possibility A	Possibility B
*Part-time work*	Yes	No	No	Yes
*Customized work*	No	Yes	Yes	No
*Extra breaks with pay*	No	Yes	Yes	No
*Medical visit work hours*	No	Yes, without pay	Yes, without pay	Yes, with pay
*Monthly pay reduction*	7 €	66 €	27 €	13 €
*Preferred option (A or B)?*	о	о	о	о
	Discrete Choice Experiment 5.		Discrete Choice Experiment 6.	
Attributes *(keywords)*	Possibility A	Possibility B	Possibility A	Possibility B
*Part-time work*	Yes	No	No	Yes
*Customized work*	Yes	No	No	Yes
*Extra breaks with pay*	Yes	No	Yes	No
*Medical visits work hours*	Yes, without pay	Yes, with pay	No	Yes, with pay
*Monthly pay reduction*	13 €	27 €	27 €	13 €
*Preferred option (A or B)?*	о	о	о	о

Finally, respondents were asked whether they were certain about their answers. If they were uncertain, they were asked why. Respondents who reported that they did not understand the scenarios or reported that they were bored with them and wanted to be done with the questionnaire were excluded from further analysis.

### Statistical analyses

Following data validation, the conditional logit model was used to analyse the discrete choice responses. The probability of choosing an alternative *j* from n_*i*_ in a choice scenario *i* can be defined as follows:

$$ P(j)=\frac{\exp \left({X}_{\mathrm{ij}}^{\prime}\beta \right)}{\sum_{k\in {C}_i}\exp \left({X}_{\mathrm{ik}}^{\prime}\beta \right)}, $$

where there are n_*i*_ = 2 possible choices in each scenario’s choice set C_*i*_ [23]. For all attribute levels, WTP was calculated by dividing the estimated coefficient (β) by the payment coefficient. This approach has an underlying rationale derived from the economic theory of demand, in which these calculated ratios are known as marginal rates of substitution [24].

WTP is calculated as a ratio of two stochastic variables, and it is not possible to derive confidence intervals (CIs) directly from the parameter estimates of the conditional logit estimations. Therefore, bootstrapping was performed. Bootstrapping assumes that the data are a random sample of the whole population. It simulates what would happen if repeated samples from the whole population were drawn, using repeated samples of available data. Empirical research suggests that the best results are obtained when the repeated samples are the same size as the original sample and when the repetition is done with replacement [25]. To derive the confidence intervals for the WTP results, 10,000 iterations were carried out [25]. *P* values <0.05 were considered significant. All statistical analyses were performed using SAS version 9.4.

## Results

### Sample characteristics

The average age of participants was 46 years (standard deviation (SD) = 10.5) for the general sample, 55 years (SD = 6.8) and for the T2D sample, and 56 years (SD = 7.1) for the matched sample. The average Body Mass Index was 26 (SD = 4.8) for the general sample; 30 (SD = 5.7) for the T2D sample; and 26 (SD = 5.8) for the matched sample. Participants from the T2D sample appeared less educated, more likely to be working part time, and more likely to be working in the public sector (Table 2). The median number of years with T2D was 7 (average = 9, SD = 7) and around one fifth of participants were insulin dependent and experienced diabetes specific complications.

**Table 2:** Characteristics of participants

	General population		T2D sample		Matched sample	
	*n* = 600		*n* = 693		*n* = 539	
	*n*	%	*n*	%	*n*	%
*Gender*						
Male	309	52	420	61	315	58
Female	291	49	273	39	224	42
*Higest obtained education*						
Primary school/short education	270	45	401	58	244	45
Medium long/long education	330	55	292	42	295	55
*Employment sector*						
Public	360	40	309	45	205	38
Private	240	60	384	55	332	62
*Type of employment*						
Full-time	574	96	577	83	498	92
Part-time	26	4	116	17	41	8
*Income level*						
Low (below 6711 €)^a^	272	45	336	48	211	39
High (above 6711 €)^a^	251	42	274	40	259	48
Prefer not to answer	77	13	83	12	68	13
*Relative or friend with diabetes?*						
No	335	62	–	–	184	66
Yes	205	38	–	–	352	34
*Diagnosed with diabetes*						
Type 1 diabetes	17	3	0	0	–	–
Type 2 diabetes	43	7	693	100	–	–
*Blood glucose lowering medicine*						
Yes	27	5	544	78	–	–
No	573	95	149	22	–	–
*Insulin treatment*						
Yes	6	1	149	22	–	–
No	594	99	544	78	–	–
*Diabetes specific complications* ^b^						
Yes	36	6	120	17	–	–
No	564	94	573	83	–	–
*Self-rated health*						
Good	443	74	375	54	410	76
Bad	157	26	318	46	129	24
*Perceived severity of T2D* ^c^						
Severe	174	29	224	34	149	28
Not severe	404	67	446	65	374	69
Don’t know	22	4	3	1	16	3

^a^Conversion from DKK (Danish Krone) to Euro is based on the average exchange in 2015 of 7.45

^b^Diabetes specific kidney complications, foot damage, eye changes or ‘other’

^c^When you think of the disease T2D how do you perceive it?

-item not surveyed in this sample

### WTP for flexible work conditions in the general sample

**Fig. 2:**
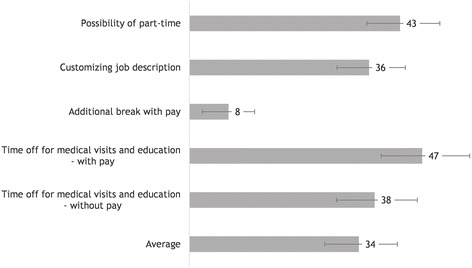
WTP in the general sample for work accommodations for people with diabetes

Figure 2 provides an overview of the preferences regarding flexible work conditions in the general sample. The results revealed WTP for all the initiatives: Average WTP for all attributes combined was 34€/month (95% CI 27–43). Average WTP for part-time work and for customized work was 43€/month (95% CI 62–97) and 36€/month (95% CI 30–44), respectively. Average WTP for time off for medical visits and patient education was higher with pay (47€/month, 95% CI 39–57) than without pay (38€/month, 95% CI 30–46). Lowest average WTP was for the attribute of extra paid breaks (8€/month, 95% CI 3–13).

Table 3 depicts WTP associated with participant characteristics. Women had a significantly lower WTP for the initiative ‘extra paid breaks’ compared to men. With the exception of gender, the WTP for the ‘part-time’ initiative varied significantly on all background characteristics: Participants who were below 50 years, had less than three years of education, had low income, worked in the private sector, had poor self-reported health, and did not perceive diabetes as a severe chronic disease, had a lower WTP compared to those that did not. Although not always statistically significant, similar trends were observed with regards to the other initiatives.

**Table 3:** WTP for work accommodations in the general sample (*n* = 600) by baseline characteristics

	Part-time work	Customized work	Extra breaks with pay	Time off with pay	Time off without pay	Average
	Average € per month	Average € per month	Average € per month	Average € per month	Average € per month	Average € per month
Average entire sample (*n* = 600)	43	36	8	47	38	34
*Gender*						
Male (*n* = 309)	43	42	17	52	43	39
Female (*n* = 291)	43	32	1	44	35	31
*P* value	0.10	0.16	**0.002**	0.40	0.35	0.09
*Age*						
25–49 years (*n* = 341)	36	32	8	38	30	29
50–67 years (*n* = 259)	54	45	7	63	50	44
*P* value	**0.03**	0.08	0.81	**0.01**	**0.03**	**0.006**
*Educational level*						
Primary or short (*n* = 270)	33	33	10	46	37	32
Medium-long or long (*n* = 330)	53	40	5	49	38	37
*P* value	**0.01**	0.30	0.35	0.75	0.88	0.30
*Employment sector*						
Private (*n* = 360)	33	37	3	45	29	29
Public (*n* = 240)	51	36	12	50	45	39
*P* value	**0.02**	0.95	0.06	0.56	**0.04**	**0.05**
*Income* ^a^						
Low (*n* = 272)	34	31	6	43	37	30
High (*n* = 251)	53	47	12	56	38	41
*P* value	**0.03**	**0.04**	0.33	0.22	0.93	0.05
*Relative or friend with diabetes*						
Yes (*n* = 335)	59	43	8	45	40	39
No (*n* = 205)	33	39	10	49	37	34
*P* value	**0.002**	0.70	0.71	0.69	0.77	0.39
*Self-reported health* ^b^						
Good (*n* = 443)	48	43	7	54	44	39
Poor (*n* = 157)	32	23	11	35	25	25
*P* value	**0.03**	**0.002**	0.49	**0.03**	**0.01**	**0.001**
*Self-reported chronic disease* ^c^						
Yes (*n* = 392)	51	40	8	46	44	38
No (*n* = 208)	30	32	8	49	28	29
*P* value	**0.002**	0.23	0.90	0.76	**0.04**	0.08
*Perceived severity of diabetes* ^d^						
Severe (*n* = 174)	65	39	13	57	37	42
Not severe (*n* = 426)	35	36	6	44	37	32
*P* value	**0.001**	0.71	0.32	0.22	0.97	0.08

Note: *P* values are for differences in WTP between variables. No adjustments for multiple testing were performed. Bolded results are statistically significant

^a^Low and high income were defined as ≤ and >6666.6 € (50,000 DKK) per month, respectively

^b^Self-reported heath was dichotomized as good (good, very good) and poor (somewhat poor, poor, very poor)

^c^Depression, asthma, atherosclerosis, diabetes, stroke, cancer, hearing loss, back pain, migraine, arthritis

^d^When you think of the disease T2D how do you perceive it'?

### WTP for flexible work conditions in the T2D sample and matched sample

In general, there was a positive WTP for the different initiatives among participants with T2D and among participants who had been matched to them (Table 4). Average WTP was generally higher in the matched sample (55 €/month) than in the T2D sample (32 €/month), a difference that was statistically significant at *p* < 0.001). WTP was also significantly higher within the matched sample with regard to part-time work, customized work, and time off with and without pay. The lowest average WTP for both groups was observed in relation to extra breaks with pay (T2D sample, 11 €/month, 95% CI 7–15; matched sample, 18 €/month, 95% CI 10–26). WTP to pay for medical visits and patient education was higher with pay than without pay in the two groups. There were no significant between-group differences in WTP for extra breaks with pay or time off with pay.

**Table 4:** WTP for work accommodations for people with diabetes in the T2D and matched samples, average € per month

	T2D sample		Matched sample		Difference
	*n* = 693		*n* = 539		Matched-T2D sample
	WTP €/month	CI	WTP €/month	CI	*P*-value
*Attributes*					
Part time work	36	[31–42]	76	[62–97]	<0.001
Customized work	28	[23–33]	61	[49–76]	<0.001
Extra breaks with pay	11	[7–15]	18	[10–26]	0.132
Time off for medical visits with pay	57	[50–64]	68	[53–85]	0.228
Time off for medical visits without pay	27	[21–34]	53	[41–69]	<0.001
Average	32	[26–38]	55	[43–71]	<0.001

*Note*: CI 95% confidence interval

## Discussion

We observed overall WTP for all initiatives providing flexible working conditions for people with diabetes among samples representing the general population, people with T2D, and participants matched by age, gender, and region to people with T2D. However, WTP for additional breaks with pay was considerably lower in comparison to the other initiatives. Compared to people without diabetes, people with T2D had significantly lower WTP for some of the initiatives (possibility of part-time job, customized work, and time off without pay). In addition, WTP in the general sample varied by participant characteristics; participants younger than 50 years, working in the private sector, and with poor self-reported health had lower WTP than participants without these characteristics. Although previous qualitative studies reported that the possibility of taking frequent breaks during the work day was an important facilitator of managing diabetes at work [11, 12, 14], our findings showed low WTP for extra breaks, particularly among people with T2D.

The participants WTP were likely to be influenced by a complex interplay between structural, environmental and psychosocial factors. Laws and practices underpinning work accommodations are among the structural factors that may contextualize the findings. In some countries, including Denmark, existing laws enable financial compensation for employers for expenses incurred in relation to all work accommodation initiatives except extra breaks with pay. In Denmark, municipalities can reimburse employers for expenses related to work accommodations due to chronic illness, such as part-time work due to permanently reduced ability to work, vocational training and retraining (work customization), and absences from work due to chronic illness (time off with or without pay). Consequently, participants may have viewed these accommodations as already falling within the remit of social responsibility; that is, something to which they were already contributing by virtue of paying taxes.

In contrast, the accommodation allowing for extra breaks is not currently included in these reimbursements and is therefore not an established social convention. Participants may have also perceived extra breaks as a need more appropriately addressed within informal work relationships than by structural intervention. Lack of formality and regulations could help explain the low WTP for extra breaks. Future research may be able to clarify whether an underpinning of regulations and formal guidelines for work accommodations aids implementation of these initiatives.

People with T2D had lower WTP than people without T2D in terms of three initiatives (part-time work, customized work, and time off without pay), but no significant differences were observed for extra breaks or time off with pay. The lower WTP for time off without pay among people with T2D might be explained by the content of the initiative; it may not make sense to individuals with T2D that they should pay for an initiative that already reduces their paid hours of work. Although we did not examine the participants’ motives for their WTP, the lower WTP with regard to part-time work and customized work among people with T2D may reflect the right to be employed on special terms due to a health-related disability. People with T2D may not be willing to pay for something that they regard as a societal responsibility and may perceive this option as an injustice. However, it may also be possible that people with T2D do not want to be employed on special terms to avoid being assigned a sickness role that can be associated with stigma, as noted by Parsons decades ago [26]. Accordingly, fear of colleagues’ and managers’ negative attributions can make it socially undesirable to be employed on special terms. Nonetheless, these explanations remain speculative; we did not directly examine the motives for their WTP preferences.

Among the strengths of this study were the relatively large samples and the use of an innovative, yet well-established method for investigating population perspectives on workplace prevention of early retirement and productivity loss due to T2D. Limitations of the study include the possibility of selection bias due to recruitment of samples from web panels. Although we included a sample that matched the T2D sample with regard to age, gender, and region, we cannot rule out that people, who participate in web based panels may vary from those who do not in systematic ways. We chose a web based panel, because we aimed to recruit a large sample of employed individuals between 25 and 67 years. Also, participants from T2D web-based panels would be more representative of a national T2D population than participants recruited from hospital based clinics. In Denmark, around 90% of people with T2D are treated by their general practitioner in primary care and 10% in specialized diabetes outpatient clinics, but only if their diabetes is complicated and not possible to treat in primary care.

Discrete choice experiments have been an effective way to determine people’s healthcare preferences. Although the proportion of unreliable responses (e.g. of respondents did not understand, were bored, or wanted to be done with the questionnaire) were relatively low in this survey (4%), discrete choice experiments’ application to complex social phenomena such as preferences for flexible work conditions may be problematic; the multi-attributable nature of these phenomena may challenge transparency. Future studies should examine the motives for WTP as these can help inform the drivers of people’s preferences. We found consistent results across groups, which strengthened validity. However, the results may primarily be generalizable to individuals with T2D in countries with similar health care and social security systems.

## Conclusion

People with and without T2D have WTP for initiatives that support people with T2D in managing their diabetes at work: part-time work, work customization, time off for medical visits with and without pay, and extra breaks with pay. WTP was generally lower in people with T2D, and WTP for extra breaks with pay was considerably lower compared to the other initiatives, possibly because it lacks a legal and economic foundation. Adequate implementation of these initiatives seems feasible and may help prevent unnecessary exclusion of people with T2D from the workplace. Future evaluations of such workplace initiatives are needed to determine their effectiveness.
